# Detection of 4977-bp deletion of mitochondrial DNA in in vitro fertilization failure women: A case-control study

**Published:** 2018-09

**Authors:** Seyed Hamidreza Mirabutalebi, Noorodin Karami, Hamid Reza Ashrafzadeh, Zhima Akhvansales, Maryam Tavakoli, Nasrin Ghasemi

**Affiliations:** 1 *Genetics Department,* *Shahid Sadoughi University of Medical Sciences, Yazd, Iran.*; 2 *Abortion Research Centre, Yazd Reproductive Sciences Institute, Shahid Sadoughi University of Medical Sciences, Yazd, Iran.*; 3 *Biotechnology Research Centre, International Campus, Shahid Sadoughi University of Medical Sciences, Yazd, Iran.*; 4 *Research and Clinical Center for infertility, Yazd Reproductive Sciences Institute, Shahid Sadoughi University of Medical Sciences, Yazd, Iran.*; * Seyed Hamidreza Mirabutalebi and Noorodin Karami are both first authors.

**Keywords:** In vitro fertilization, Mitochondria, Mutation, Cell aging

## Abstract

**Background:**

The quality of oocyte is often considered as a limiting factor for fertility, especially IVF. Some mitochondrial mutations, particularly the 4977-bp deletion increase with the age. Thus, this mutation can serve as a marker for cell aging, which indicates the reduced quality of the oocytes for fertilization. It has been suggested that this can also be investigated in the blood cells of women with IVF failure.

**Objective:**

1-Determination of the frequency of 4977-bp deletion in women with IVF failure, 2-Investigation of the relationship between 4977-bp deletion and the age of patients.

**Materials and Methods:**

Polymerase chain reaction was used to detect the 4977-bp deletion in blood samples of 52 IVF failure women and 52 women who had at least one healthy child. After polymerase chain reaction with deleted and wild-type primers, the products were examined using agarose gel electrophoresis.

**Results:**

48.07% of women with IVF failure and 34.62% of healthy women had a mitochondrial 4977-bp deletion, with p=0.163 and OR: 1.749. Also, in association with the age of these patients and the frequency of 4977-bp mutation, p and OR were obtained 0.163 and 1.749, respectively and frequency of this mutation was higher in patients over 35 yr old compared to other subgroups (Patients ≥35: 57.69).

**Conclusion:**

According to the findings of this study, there is no a significant relationship between the frequency of mitochondrial 4977-bp mutation and failure in IVF.

## Introduction

Infertility involves approximately 10-15% of couples (1). In the young and healthy people, the possibility of pregnancy in each reproductive cycle is usually 20-25% and during a year is about 90% (2). Indeed, a standard definition of clinical infertility is one yr of unprotected intercourse without pregnancy (3). The effect of maternal age on reducing fertility and increasing the pregnancy loss cannot be ignored (4). In spite of the causes of infertility, in vitro fertilization (IVF) is the treatment that results in the highest fertility rate per cycle. Frequent failure in IVF is usually defined as a failure in pregnancy after 2-6 cycles of IVF (5, 6). There are several reasons for failure of fertilization after IVF. Sperm defect (7), the interaction of defective sperm and the zona pellucida (8) and abnormalities in the oocyte have all been reported as potential causes of failure in fertilization (9, 10). 

Also, the oocyte quality is always considered as a limiting factor in fertility, especially IVF, which is often influenced by age. The quality of the oocyte is affected by two important factors: nuclear maturity and cytoplasmic maturity (11). Nuclear maturity occurs when the first polar body is seen, but the cytoplasmic maturation of the oocyte is not visible and one of the important factors involved in this occurrence is mitochondria (12). Mitochondria are the only semi-autonomous cytoplasmic organelles and have their own specific genome (13). These organelles have different roles in the cell, including as only ATP-supplied cellular organelle that produces energy during the process of fertilization and replacement of the fetus (14). Also, mitochondria are a source of intracellular calcium reserves and factors involved in apoptosis (15). 

Cell aging is a degenerative process associated with the progressive accumulation of adverse changes during the time, reduced physiological function, and increased risk of death (16). Some studies showed a wide range of mitochondrial changes and mitochondrial DNA (mtDNA) associated with cell aging, including mitochondrial dysfunction, decreased oxidative mitochondrial phosphorylation, accumulation of mtDNA mutations, increased mitochondrial production of reactive oxygen species, and increased oxidative damage to DNA, proteins, and lipids (17-19). 

The 4977-bp deletion mutation of mtDNA (ΔmtDNA4977) occurs between 8469 and 13,447 nucleotides and in many diseases, its association with age has been reported (20, 21). In the tissues that are not divided, this mutation can serve as a marker for the study of the cell aging, including oocytes, which indicate the reduced quality of the oocytes for fertilization. Oocytes, due to the increased probability of cell aging in them (20, 22), are suitable cells for the study of 4977-bp deletion mutation, but some studies have proven that these can also be studied in blood cells (23). 

Therefore, the association between the frequency of the 4977-bp deletion and IVF failure in blood samples of patients with IVF failure was examined.

## Materials and methods

**Patients**

In this case-control study, all patients referred to the Yazd Research and Clinical Center for Infertility from September 2016 to March 2017 who had two or more IVF failures and their partners had normal spermograms, were randomly studied.

The women with history of smoking, chromosomal abnormalities, recurrent miscarriage, autoimmune diseases, reproductive disorders, anti-phospholipid syndrome, diabetes mellitus, thrombo-embolic events, uterine cavity abnormalities, thyroid dysfunction, male factor infertility and endometriosis were excluded from the study. The criterion for considering repeated IVF failure was at least two consecutive failed IVF cycles. These failed cycles were described as failure to achieve clinical pregnancy in a cycle, in which at least three well-quality embryos with grade I or II were transferred.

Healthy people were also selected from women who had at least one healthy child and matched in terms of age with the patient group. The sample size in each group was 52, and in each group, subjects were divided into two subgroups less than 35 yr old and over 35 yr old. There were 26 people in each subgroup (Table I). At first, 5 ml of complete blood was taken from all subjects and stored in the CBC tubes containing an anti-coagulant EDTA. After the blood sampling of all the people in the study, blood samples were stored at -20^o^C until extraction.

**Total DNA extraction and polymerase chain reaction**

At first, Total DNA was isolated from the blood of healthy and patient individuals by the genomic extraction kit (Genet Bio), according to its instructions, and the PCR reaction was performed using two pairs of primers. These primers were taken from Thayer *and colleagues* (24). The sequence of the primers is shown in table II.

A pair of primers (Deleted) was linked to the two sides of the deleted area, therefore, only in individuals with a deletion area were amplified. The other pair of primers (Wild-type) was to confirm the presence of mtDNA, therefore it was amplified in all individuals. The PCR reaction was carried out in a final volume of 25 μl containing 100 ng of genomic DNA, 10 pmol of each pair of each primer, 0.2 mM of each deoxy-nucleotide triphosphate, 20 mM of Tris-HCL buffer at pH=8.3, 50 mM KCL, 3 mM MgCl2 and 1.25 units of Taq polymerase enzyme. In the first cycle, the denaturation temperature was 94^o^C and for 10 min (hot start), and then in the next 30 cycles, the same temperature was applied for 1 min. Also, the annealing temperature was 67^o^C for 30 sec, and finally the extension phase temperature was about 72^o^C for 30 sec, while the same temperature was maintained in the final phase for 5 min. Consequently, the PCR products were separated by agarose gel (2%) along with ethidium bromide staining.

**Ethical consideration**

All samples were taken after receiving informed consent from the patient and the approval of the Research and Clinical Center for Infertility (code number: 2813).

**Statistical analysis**

Data were collected based on the results of the experiments and analyzed using SPSS software (Statistical Package for the Social Sciences, version 24.0, SPSS Inc, Chicago, Illinois, USA (SPSS). Chi-square and Odd Ratio tests were used to study the relationship between the variables and the significance level and confidence intervals (CI) were considered p<0.05 and 95%, respectively.

**Table I:** Different subgroups of subjects participating in the study (n=26)

**Statistical society**	**under 35 yr old**	**over 35 yr old**
History of two or more IVF failure	29.6 ± 3.1	38.4 ± 1.2
healthy women	28.3 ± 5.2	37.6 ± 2.4

**Table II:** The sequence of the primers of deleted and wild-type

**Primer pair**	**Forward**	**Reverse**	**length product**
Wild-type	5ʹ-TATACCGCCATCTTCAGCAAAC-3ʹ	5ʹ-TACTGCTAAATCCACCTTCGAC-3ʹ	177-bp
Deleted	5ʹ-CCTTACACTATTCCTCATCACC-3ʹ	5ʹ-TGTGGTCTTTGGAGTAGAAACC-3ʹ	127-bp

## Results

**Figure 1:**
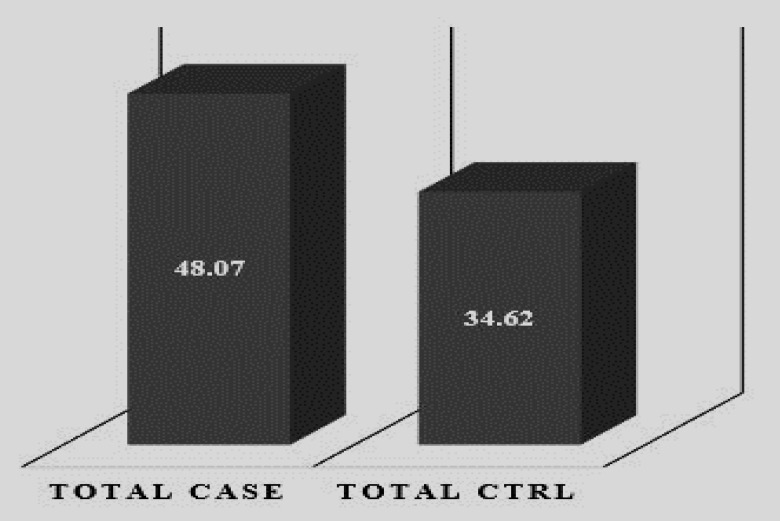
The comparison of the frequency of 4977-bp deletion between women with IVF failure and healthy controls

**Figure 2:**
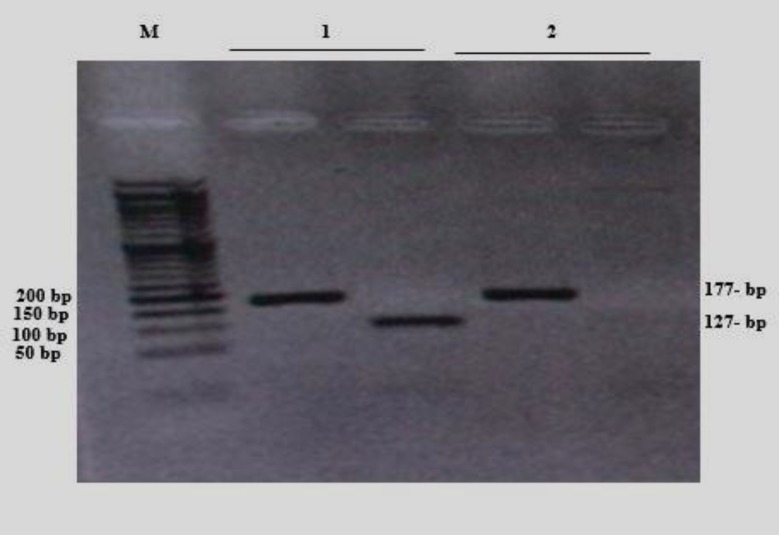
The detection of 4977-bp deletion. Lane M: molecular marker, 1 showing a patient with 4977-bp deleted mtDNA and 2, a healthy control with wild-type mtDNA, 127-band: deleted mtDNA, 177-band: wild-type mtDNA

In the present study, 48.07% of patients had a 4977-bp deletion, while in healthy controls, the rate of this mutation was 34.62% (Figure 1). The bands with a length of 177-bp represent the presence of mtDNAs and the bands with a length of 126-bp show 4977-bp deletions (Figure 2).

**Figure 3:**
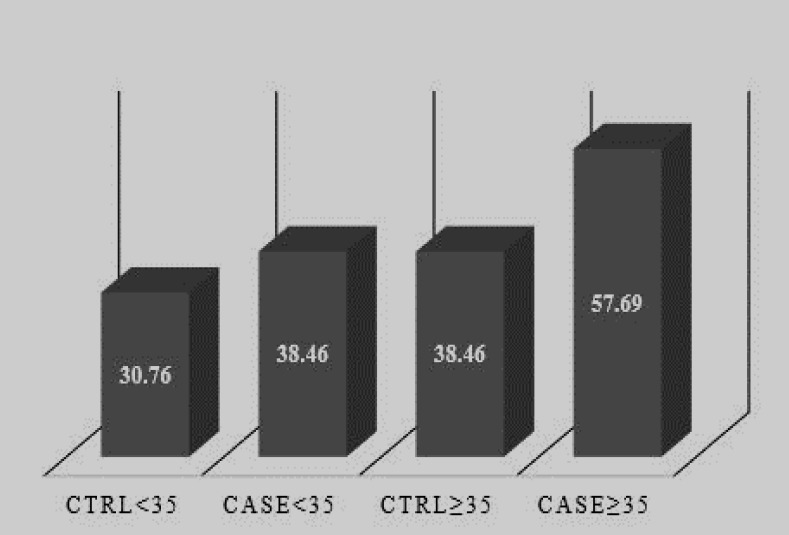
The comparison of the frequency of 4977-bp deletion among subgroups

Also, Odd Ratio (OR) and significance level were obtained at 1.749 and 0.163, respectively. The rate of this mutation among subgroups is as follows:

Patients ≥35: 57.69%, patients <35: 38.46%, control ≥35: 38.46%, control <35: 30.76% (Figure 3). Therefore, the highest frequency is for patients over 35 yr old. In addition, there was no a significant correlation between the age of patients and the frequency of 4977-bp deletion (case and control over 35 yr old: p=0.165 and OR: 0.45, case and control under 35 yr old: p: 0.56 and OR: 0.34).

## Discussion

Failure in IVF is affected by several factors, that one of the most important factors is the oocyte quality (25). The oocyte quality can be defined as its abilities to be fertilized and is controlled through mitochondria (26). Since mitochondria provide the necessary energy for the fertilization process, and each damage to mtDNA may affect this process (27). One of the most prevalent mutations of the mtDNA is the 4977-bp deletion which eliminates a segment of DNA that contains encoding genes ATPase6, ATPase8, cytochrome oxidase III, 3-NADH-dehydrogenase (ND3), ND4, ND4 subunit L (ND4L), and ND5 (28). Some genes are involved in the respiratory chain and can reduce cellular energy (as ATP) (29). 

Therefore, in cells that have this mutation, there are no oxidative phosphorylation genes, which leads to a general decrease in cellular energy. Hence, this mutation may lead to failure in fertilization. The association of this deletion with many diseases has been confirmed, including the study of Gashti and his colleagues who examined the 4977-bp mutations in the semen samples of varicocele patients. In this study, the frequency of this mutation was found to be 81.66% of patients (30). Also, in the study of Salehi and his colleagues on the tissue of endometriosis, the frequency of these mutations was 60% and the association of these mutations with endometriosis was reported positive (31). 

In our study, which was performed in the blood samples of women with IVF failure, the frequency of mutations of 4977-bp in patients and in healthy controls was 48.07% and 34.62%, respectively, and the relationship between this deletions and failure rate in IVF cycles was not significant. The decreased mitochondrial function is correlated with related-age changes (32). Mitochondrial mutations, which alter the expression of genes involved in oxidation phosphorylation complexes, can lead to mitochondrial dysfunction and increased production of reactive oxygen species (33). Many studies have shown that with advanced age, the rate of 4977-bp mutations also increases (34, 35). 

Also, Chan and co-workers compared the two parameters of deletion and the age of the patients in 155 unfertilized oocytes from 52 patients and the incidence of deletions in these oocytes was 34.6%, and in women older than 35 yr old, a higher incidence of these mutations were observed (36). However, some studies have not confirmed this relationship (37, 38). In our study, despite the relatively high frequency of 4977-bp mutations in individuals over 35 yr old, compared to those under 35 yr old, the association of these mutations with advanced age was not significant.

## Conclusion

Therefore, as a general conclusion, despite the relatively high frequency of 4977-bp mutations in women with IVF failure, there was no significant association between these mutations and the failure in IVF as well as the age of subjects with failure in IVF in blood samples and it is better to these mutations investigate in the oocytes of these patients and in a larger statistical population.
